# Patient-Reported Outcome Measures in High-Risk Medical Device Registries: A Scoping Review

**DOI:** 10.1093/asjof/ojae015

**Published:** 2024-03-16

**Authors:** Michelle Merenda, Arul Earnest, Rasa Ruseckaite, Wai Chung Tse, Elisabeth Elder, Ingrid Hopper, Susannah Ahern

## Abstract

Little is known about the methods and outcomes of patient-reported outcome measure (PROM) use among high-risk medical device registries. The objective of this scoping review was to assess the utility and predictive ability of PROMs in high-risk medical device registries. We searched Ovid Medline, Embase, APA PsychINFO, Cochrane Library, and Scopus databases for published literature. After searching, 4323 titles and abstracts were screened, and 262 full texts were assessed for their eligibility. Seventy-six papers from across orthopedic (*n* = 64), cardiac (*n* = 10), penile (*n* = 1), and hernia mesh (*n* = 1) device registries were identified. Studies predominantly used PROMs as an outcome measure when comparing cohorts or surgical approaches (*n* = 45) or to compare time points (*n* = 13) including pre- and postintervention. Fifteen papers considered the predictive ability of PROMs. Of these, 8 treated PROMs as an outcome, 5 treated PROMs as a risk factor through regression analysis, and 2 papers treated PROMs as both a risk factor and as an outcome. One paper described PROMs to study implant survival. To advance methods of PROM integration into clinical decision-making for medical devices, an understanding of their use in high-risk device registries is needed. This scoping review found that there is a paucity of studies using PROMs to predict long-term patient and clinical outcomes in high-risk medical device registries. Determination as to why PROMs are rarely used for predictive purposes in long-term data collection is needed if PROM data are to be considered suitable as real-world evidence for high-risk device regulatory purposes, as well as to support clinical decision-making.

**Level of Evidence: 4:**

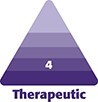

High-risk medical device registries provide high-quality, uniformly collected data to monitor healthcare processes and outcomes relating to the use of high-risk implantable medical devices. Recent high-profile device failures, such as the Poly Implant Prothèse and Johnson & Johnson pelvic mesh scandals, underscore the need for high-quality information on the postapproval performance of medical devices used in clinical care.^1-4^ High-risk medical devices, classified as a Class III or above medical device, are associated with complications, including but not limited to biocompatibility failure, device failure leading to revision surgery, and unexpected, infrequent complications, such as the metal-on-metal hip joint, causing cobalt metallosis and cardiomyopathy, or in the case of breast implants, breast implant-associated anaplastic large cell lymphoma.^5-8^ High-risk medical device registries undertake long-term monitoring and surveillance of high-risk medical devices in vivo, often for decades, in order to identify their effects on health. Device manufacturers are required to demonstrate device safety and efficacy in order to obtain market approval, but high-risk device registries are capable of capturing robust, real-world data beyond that which individual manufacturers can capture.^4^

Patient-reported outcome measures (PROMs) are emerging as a valuable source of patient outcome data and are increasingly being used as a health outcome tool by clinical registries. In the form of a questionnaire completed by patients, PROMs convey important information about a patient's health.^9-11^ The anticipated benefits of using PROMs in clinical registries include monitoring of the patient journey over time, assessment of functional outcomes, and measurement of processes of care and overall intervention effectiveness.^12^ Additionally, shared decision-making is fast becoming the optimal model on which clinical decisions are made.^9^

In high-risk medical device registries, the systematic gathering and recording of data have previously relied on clinical outcomes alone. Transcatheter heart valve thrombosis following transcatheter aortic valve replacement or stress shielding of the proximal femur following hip implant surgery are examples of previously studied clinical outcomes that require clinical diagnosis independent of patient perspective. However, clinical outcomes alone may not constitute sufficient evidence for clinical decision-making, as they do not consider the nuances of the patient journey.^9^ In addition, clinician follow-up may only be for short periods of time; thus, registries that collect PROMs could potentially enable longer term individual follow-up. By providing additional information to that of the clinician, PROMs counter the issue of selective reporting and favorable research.^10^ However, like clinical data, PROMs may be biased either positively or negatively themselves, and PROM results should be interpreted with caution. There are challenges that registries experience when collecting PROMs, such as determining the minimum response rate (RR) required to draw meaningful conclusions, and from a health economic viewpoint, the resources and monitory outlay vs response. Nevertheless, when PROMs are used to supplement clinical data, the coevaluation between patient and surgeon can facilitate improved health outcomes that are congruent with the values of both parties, over clinical data alone.

Introduced in 2014, the Australian Breast Device Registry (ABDR) is a high-risk clinical device registry that was established to accurately measure health outcomes after the implantation of breast devices.^13^ The ABDR implemented registry-wide PROMs in 2017 with the primary aim of assessing the use of PROMs to identify poorer breast device performance.

Because of the paucity of knowledge surrounding the role of PROMs for high-risk devices, we sought to scope the literature to understand the utility and predictive ability of PROMs in high-risk medical device registries. Given the primary aim of PROMs for the ABDR, particular focus was placed on studies that used PROMs as a predictive factor for clinical or quality of life outcomes.

## METHODS

As the scope of the literature in this field was novel, a scoping review was chosen to identify the extent of existing literature and existing knowledge gaps. Scoping reviews are defined as a type of systematic evidence synthesis that aims to present an overview of a potentially large and diverse body of literature, unlike a systematic review which collates empirical evidence on a smaller more focused research question.^14^ As the phenomenon of interest was not the outcome of each study, but rather, the way in which the PROMs were used, a quality appraisal of the studies was not conducted. Further, as per the Joanna Briggs (JBI) Manual for Scoping Reviews, a quality assessment is not a required element of scoping reviews.^15^ This review was undertaken using the JBI scoping review methodology.^15^

Key questions to guide data extraction were:

1. Which type of device registry uses PROMs?
2. How are PROMs used in the study design for these registries?
3. What are the PROMs time points used for these device registries (eg, short or long term)?
4. What were the PROM RRs, including at different time points?

### Information Sources

We searched Ovid Medline, Embase, APA PsychINFO, Cochrane Library, and Scopus electronic databases for the published literature. A date range was not imposed when conducting the database searches. The last search was conducted on January 16, 2022. For each article selected for inclusion, abstracts and full articles were obtained. Articles were restricted to peer-review and those written in English. We considered a high-risk device registry to be a registry that captured information on a surgical insertion of a Class III device (as per US FDA Medical Device Product Classification page or TGA)^16^ (Table 1). The following publications were excluded as they did not match the concept of the research question: insertion of a Class II device or lower; studies where PROMs are not a main focus of the study (eg, focus on clinical outcomes); nondevice implant registries (eg, cancer, organ transplant registries); PROMs administered to anyone other than the patient; single case studies; 50 or fewer study participants; psychometric or feasibility studies; and gray literature. The decision to exclude gray literature was made to ensure the integrity and authenticity of the articles; and, after the data extraction process, the contributions of gray and white literature are hard to discern.^17^

**Table 1.** Medical Device Product Classification

Medical device classification	Risk	Definition (FDA)	Example
Class I	A medical device with low-to-moderate risk that requires general controls	Not intended for use in supporting or sustaining life Do not present a potential unreasonable risk of illness or injury	Oxygen mask Bandages Tongue depressor Reusable surgical scalpel
Class II	A medical device with a high-to-moderate risk that requires special controls	Devices for which general controls are insufficient to provide reasonable assurance of the safety and effectiveness of the device	Catheters Sutures Contact lenses Syringes
Class III	A medical device with high risk that requires premarket approval	Usually, sustain or support life is implanted or present a potential unreasonable risk of illness or injury	Breast implants Pacemakers Implanted prosthetics Defibrillators

### Eligibility Criteria

Quantitative studies (eg, registry, cohort, longitudinal, prospective, and retrospective), describing the use of PROMs within a high-risk device registry or database context, were included. The main phenomena under investigation were the utility of PROMs in high-risk medical device registries and included recipients of Class III medical devices. Organ implant and resorbable implant device registries were excluded. The topics of focus for this review included device registry type, PROM tool, follow-up time, and type of statistical analysis undertaken. Further exclusion criteria were applied during the full text screening process. Articles were excluded for the following reasons: abstract only, not a device registry, could not locate full text, not relevant, duplicate, no PROMs, single surgeon registry, not a study, clinical outcomes, study design, resorbable implant, non-Class III medical device, psychometric studies, registry/database is unclear. This study aimed to review registry-based studies and, therefore, randomized controlled trials (RCTs) were excluded.

### Search Strategy

A systematic search of the literature was carried out to identify publications from high-risk device registries that utilized PROMs. The search strategy was developed by 2 authors, M.M. and I.H. Medical subject headings (MeSHs), keywords, and free-text search terms were used. The databases were chosen after referring to publications describing “optimal database combinations” for literature searches in the biomedical and health sciences.^18^ The following key words were searched: Patient Reported Outcome Measures/or “Surveys and Questionnaires”/or patient reported outcome measure*.mp. or Outcome AND device.mp. or implant.mp. or prosthes*.mp. AND registry.mp. or Registries/database.mp. Key terms were mapped to MeSHs and then exploded to yield the most relevant indexed articles. We adapted the search strategy to the remaining databases mentioned above. The terms were combined through the process of Boolean operators.

### Data Management

Records identified through database searching were exported to Endnote reference manager (Clarivate, London, United Kingdom), and duplicates were removed.^19^ The remaining references were then exported to Covidence (Melbourne, Victoria, Australia), a software program that allows the manageable screening of citations.^18^

### Study Selection and Data Extraction

Titles and abstracts were screened by 2 researchers, M.M. and W.C.T., and records were excluded based on inclusion and exclusion criteria. The remaining articles underwent full text screening for eligibility by M.M. and W.C.T. Any uncertainties regarding inclusion were referred to the third researcher, S.A., for eligibility consensus. The data were extracted using a standardized data extraction spreadsheet in Microsoft Excel (Microsoft, Redmond, WA). In order to address the scoping review questions, the following data were extracted: registry name, registry type, number of study participants, PROM used in order to understand their predictive ability: how the PROMs were used in the study design (the way in which the PROMs were statistically analyzed through the statistical test/s used); PROM follow-up time points; and PROM RR as reported by the authors. As a focus was placed on the predictive ability of PROMs, we were looking for study designs that utilized regression analysis to predict clinical outcomes; compared PROMs at different time points or among different groups.

## RESULTS

**Figure 1.**
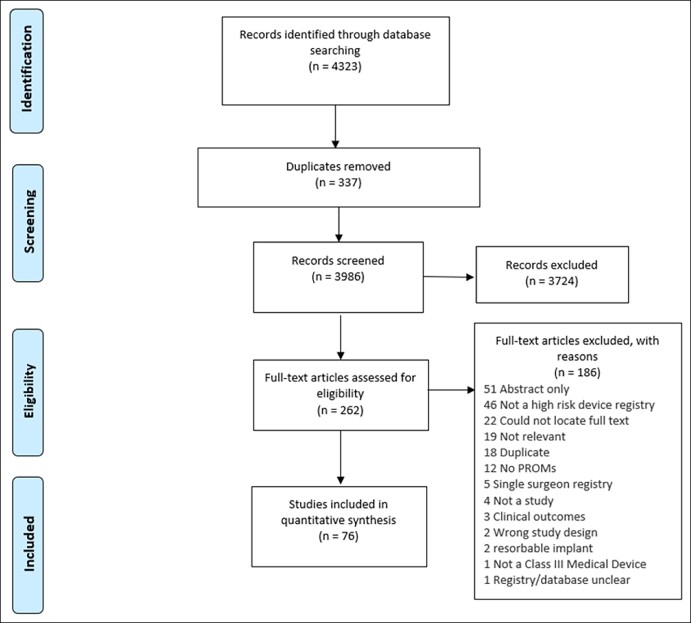
Preferred reporting items for systematic reviews and meta-analysis flow diagram patient-reported outcome measures in high-risk medical device registries.

Out of the retrieved 4323 articles, 76 fit inclusion criteria (Figure 1). Eleven papers were published between 1997 and 2010, 44 articles between 2011 and 2017, and the remaining 21 between 2018 and 2022. Interestingly, the greatest number of articles pertaining to PROMs in high-risk medical device registries were found between 2011 and 2017, more than double those for the period 2018 to 2022. The majority, 45 articles, were published in Europe and the UK,^20-68^ Oceania 10,^69-78^ North America 13,^79-91^ Asia 1,^92^ and global 3.^91-93^ Thirty-one high-risk device registries were identified from across 4 different registry types orthopedic 22 (70%), cardiac 7 (22%), hernia mesh 1 (3%), and penile 1 (3%). The number of publications per registry type were orthopedic 64 (84%),^20-64,69-86,92^ cardiac 10 (13%),^65-67,87-91,93,94^ hernia mesh 1 (1.5%),^95^ and penile 1 (1.5%).^68^

### Registry Type and Participant Numbers

Four high-risk device registry domains were identified, orthopedic, cardiac, penile, and hernia mesh. The majority of studies were related to orthopedic registries.^20-64,69-86,92^ Participant numbers varied; the largest study cohort was from the National Joint Registry for England, Wales, Northern Ireland, and the Isle of Man.^34^ The largest cardiac cohort was from the Society of Thoracic Surgeons/American College of Cardiology Transcatheter Valve Therapy Registry in the United States of America.^91^ Both the hernia mesh and penile registries studied cohorts of less than 1000 participants.^68,95^ The smallest cohorts were from the Swedish Ankle Registry^50^ followed by the Spine Tango Registry^63^ with 69 and 100 participants, respectively.

The number of participants described in the studies ranged from 69^50^ to 479,353^34^ for orthopedic, 140^65^ to 45,884^91^ for cardiac, 583^68^ for penile, and 956 participants for hernia mesh.^95^ Forty-six (60%) papers captured a population of greater than 1000 participants.

### Patient-Reported Outcome Measures

**Figure 2.**
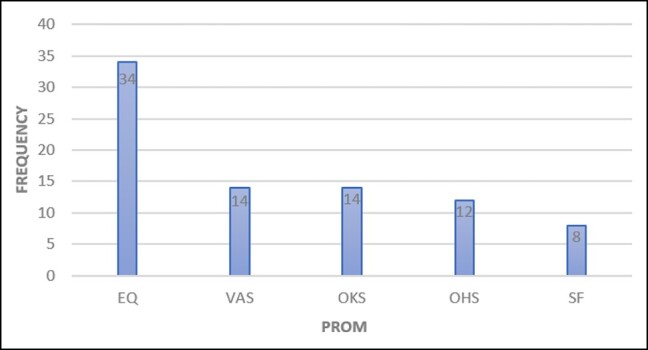
Top 5 PROM use—orthopedic registries. EQ, Euro Qol scale questionnaire; OHS, Oxford hip score; OKS, Oxford knee score; PROM, patient-reported outcome measure; VAS, visual analog scale; SF, short form survey.

**Figure 3.**
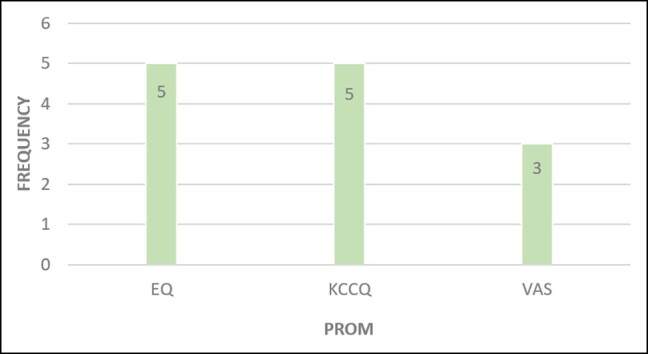
Top 3 PROM use—cardiac registries. EQ, Euro Qol scale questionnaire; KCCQ, Kansas city cardiomyopathy questionnaire; PROM, patient-reported outcome measure; VAS, visual analog scale.

Variation existed in the PROMs used across the registries, with over 22 different PROMs being used by the orthopedic registries and 9 different PROMs used across the cardiac registries. The most frequently described PROM overall was the Euroqol 5-Dimensional Questionnaire (EQ-5D), a metric for health-related quality of life and used for both orthopedic (*n* = 34)^24,26-28,31,32,35-41,43-62,64^ and cardiac registries (*n* = 5)^66,67,88,93,94^ (Figures 2, 3). Cardiac registries also frequently used the Kansas City Cardiomyopathy Questionnaire (*n* = 5)^87-91^ which measures symptoms and physical function (Figure 3). This was followed by the visual analog scale (VAS) for both orthopedic and cardiac registries, which measures pain (*n* = 14^28,43-47,50-52,54-58^ and *n* = 3,^67,88,89^ respectively). The Oxford knee score (*n* = 14)^30-34,40,41,64,73-75,77,85,92^ and Oxford hip score (*n* = 12)^28,35-39,69,70,75,76,78,85^ were also used frequently by orthopedic registries. Beyond these tools, there was wide variation in tools used.

### PROM Utility: Study Design Classifications

Four main study designs for using PROMs were described (Table 2). The majority of papers could be classified as comparative group studies (*n* = 45, 59%), that is, the outcome of the study compared the means of 2 groups, for example, it compared different devices or different surgical techniques. Another study design identified was comparison time point studies (*n* = 13, 17%), where the outcome of these studies was to compare outcomes at 2 time points, for example, comparing the means before and after surgery, or outcomes at 6 months vs at 1 year.^25,27,30,34,52,58,60,62,64,66,67,80,89^

**Table 2.** Classification of Study Utility/Study Design

Registry type	Comparative groups (cohort or surgical approach), *n* (%)	Before/after intervention comparison, *n* (%)	Predictive, *n* (%)	Time to event (survival analysis), *n* (%)
Overall	45 (59)	13 (17)	15 (20)	3 (4)
Orthopedic	40 (62)	10 (16)	13 (20)	1 (2)
Cardiac	3 (30)	3 (30)	2 (20)	2 (20)
Penile/mesh	2 (100)	—	—	—

The study design most pertinent to PROMs as predictors of outcomes was the predictive or risk factor design. This design investigated the association between potential predictive factors and outcomes, usually through a regression analysis. This was used by 15 (20%) of the studies.^21,22,28,33,40,48,51,61,71,72,75,83,86,88,90^ Eight of these 15 papers treated PROMs as an outcome only and sought to understand what clinical factors affect PROM scores, and/or used regression to understand how early PROMs were associated with longer term PROMs^21,33,40,48,51,61,83,90^; and 2 papers treated PROMs both as a factor in regression analysis and as an outcome.^28,86^ A fourth design identified was time-to-event studies, which used survival analysis to investigate outcomes, although this was used in only 3 (4%) papers.^44,87,91^ Two papers treated PROMs both as a factor in regression analysis and as an outcome.^28,86^

### PROMs as Predictive Factors for Clinical Outcomes

A subset of 5 papers out of the 15 predictive papers pertained to the use of PROMs as a predictive factor for clinical outcomes^22,71,72,75,88^ (Table 3). These 5 papers were classified based on the use of regression analysis, with PROMs built into the statistical model as a risk factor. Of the 5 predictive papers, 4 were from orthopedic registries^22,71,72,75^ (3 from the New Zealand Joint Registry^71,72,75^ and 1 was from a cardiac registry^88^). In these papers, the association between PROM outcomes and pain; implant failure rate; revision surgery; and mortality was studied. Gupta et al^71^ retrospectively looked at 6-month OSS PROM scores with respect to their relationship with revision surgery 2 years from the PROM date and concluded that poor OSS at 6 months is an independent risk factor for early revision after anatomic total shoulder arthroplasty, reverse total shoulder arthroplasty, and shoulder hemiarthroplasty. Flint et al^88^ looked to characterize the association of pre- and postimplant health status scores with outcomes following left ventricularassist device (LVAD), the authors found that preoperative health status had limited association with outcomes after LVAD; however, it found that persistently low health status after surgery may independently signal higher risk for subsequent death. Bjørnholdt et al^22^ investigated the prevalence, characteristics of, and risk factors for persistent pain 1- to 2 years after shoulder replacement and found that severe pain during the first postoperative week increased the risk of persistent pain. Rothwell et al^75^ used their study to determine the relationship between the Oxford hip score (OHS) and Oxford knee score (OKS) and the risk of early revision and reported that for every 1-unit decrease in the Oxford score, the risk of revision within 2 years increased by 9.7% for total hip replacement (THR), 9.9% for total knee replacement (TKR), and 12.0% for unicompartmental kneereplacement (UKR) (*P* < .001), and that 70% of the revisions within 2 years for TKR and 67% for THR and UKR would have been captured by monitoring the lowest 22% and 28%, respectively, and concluded by saying that the OHS and OKS 6-month scores are useful predictors of early revision after THR and TKR. Hosman's paper evaluated early results of a nationwide series of total ankle replacements performed using second- and third-generation implants. The cumulative 5-year failure-free rate was 65% for patients with an unfavorable score and 95% for those who had a favorable patient score and that each 1-point increase in the patient score (ie, poorer outcome) corresponded to a 5% relative increase in the risk of failure (*P* < .05).^72^

**Table 3.** Registry PROM Follow-up Time Points and Response Rates for the 5 Predicative Papers

Registry	Author	Time point	Response rate
New Zealand Joint Registry (New Zealand Arthroplasty Registry)	Gupta 2021^71^	6 months	68.5% (anatomic shoulder) 60.5% (reverse total) 63.5% (hemiarthroplasty)
Interagency Registry for Mechanically Assisted Circulatory Support (INTERMACS) (USA)	Flint 2017^88^	3 months	Baseline KCCQ 58%, VAS 57% 3 months KCCQ 59%, VAS 58%
Danish Shoulder Arthroplasty Registry	Bjornholdt 2015^22^	14-26 months	78%
New Zealand Joint Registry	Rothwell 2010^75^	6 months and 5 years	75% registry response rate (PROMs only sent to a randomized 20% of yearly THR and TKR recipients)
New Zealand Joint Registry	Hosman 2007^72^	6 months	74%

KCCQ, Kansas city cardiomyopathy questionnaire; PROMs, patient-reported outcome measures; VAS, visual analog scale.

### PROM Time Points

**Figure 4.**
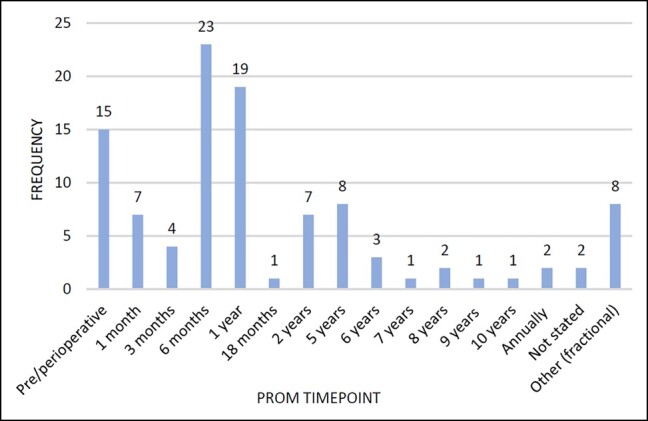
Orthopedic patient-reported outcome measure follow-up time points.

**Figure 5.**
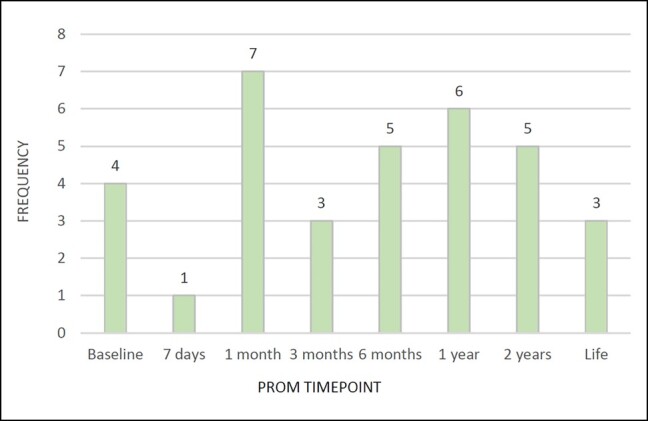
Cardiac patient-reported outcome measure follow-up time points.

PROMs were collected predominantly at 1, 2, and 5 years for orthopedic registries, and in cardiac registries up to 2 years after surgery with variation thereafter. Only 10 orthopedic papers studied PROMs at time points beyond 5 years,^40,44,47,51,54,56,58,61,62,64^ and 3 cardiac studies followed participants beyond 2 years^87-89^ (Figures 4, 5). Table 3 shows follow-up time points and RRs for the 5 predictive factor papers.

Follow-up time points for penile implants were 1 year^68^ and 1, 6, 12, and 24 months for the International Hernia Mesh Registry.^95^ Other time points consisted of fractional time points, for example, 2.5, 7.3, etc.^23-25,43,47,64,81^

### Response Rates

**Figure 6.**
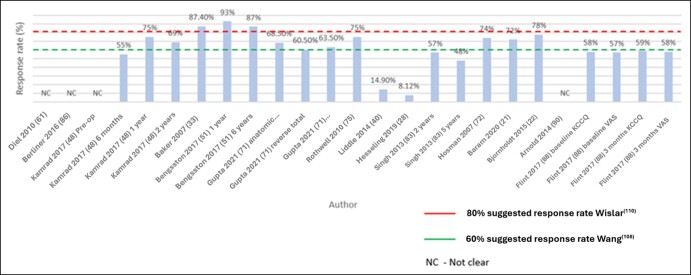
Response rates plotted against suggested registry patient-reported outcome measures minimum response rates for the 15 predictive papers. KCCQ, Kansas city cardiomyopathy questionnaire; VAS, visual analog scale.

The RRs were reported in 43 (56%) of papers.^20-23,25-28,33,34,38,40,42-54,56-59,64,68,71-73,75,76,78-80,82,83,88,95^ The overall RRs reported ranged from 8.12%^28^ to 97%^95^ RRs were unclear or insufficiently described in 14 (18%) papers.^31,32,35,36,39,61,62,68,73,85,87,89-91^ Only 60% of orthopedic studies reported an RR.^20-23,25-28,33,34,38,40,42-54,56-59,64,71-73,75,76,78-80,82,83^ Of the 10 cardiac papers, 4^65,67,93,94^ failed to report an RR and 5^66,87,89-91^ were unclear or failed to report the RR adequately, similar to the findings of others, for example, Sokas.^96^ Overall, there was high variation between the RRs that were reported for the orthopedic registries, with the lowest being 8.12%^28^ and the highest being 97%.^95^ Of the 5 predictive papers, 2^22,71^ did not discuss the use of sensitivity analysis. In a study, Gupta et al^71^ described 3 arms: total shoulder arthroplasty, hemiarthroplasty, and reverse total shoulder arthroplasty and reported the RRs as 68.5%, 63.5%, and 60.5%, respectively. Flint et al^88^ adopted inverse propensity weighting to minimize bias from missing data but did not present a sensitivity analysis. Rothwell et al^75^ described a 75% registry RR while noting that PROMs are only sent to a randomized 20% of yearly THR and TKR recipients, and Hosman et al^72^ a 74% RRs. Three orthopedic publications reported that PROM RRs decreased with increased follow-up time.^51,82,83^ In contrast, Rolfson et al,^57,58^ also orthopedic, reported RRs that increased from the preoperative time point to the 1-year time point. Other orthopedic papers in our review that collected PROMs at multiple time points did not report multiple RRs respective to the time points clearly or did not report them at all. One cardiac, INTERMACS Registry study reported RRs for different PROMs and for multiple time points and explained reasons for missing data, baseline, and 3-month RRs were below 60%.^88^ RRs reported by the International Hernia Mesh Registry indicated that they waned with increased follow-up time.^95^ RRs for Berliner et al^86^ were not stated; and Hesseling et al^28^ reported that 8.12% had OHS data completed at all time points. Refer to Figure 6 for a graph charting RRs plotted against suggested registry PROM minimum RRs for the 15 predictive papers.

## DISCUSSION

We scoped the literature to investigate the use of PROMs in high-risk medical device registries. We identified 76 papers from registries from 4 domain areas, notably orthopedic and cardiac. The most used PROMs were generic PROMs. The results highlighted that PROMs were used primarily as an outcome measure in high-risk medical device registries. We identified a small number of studies where PROMs were used as a predictive (risk) factor.^22,71,72,75,88^ In these studies, early PROMs had a high correlation with later PROMs or with an increased need for clinical intervention. PROM follow-up time points were most commonly within 2 years, with a small number of registries reporting on PROMS out to 5 years. RRs were generally reported as high, with most well over 50%, although a large proportion of studies do not report an RR. No studies reported using PROMs for prediction of device failure.

The breast device space was not represented in the literature. Orthopedic and cardiac registries represented the majority of high-risk medical device registries with the majority of them from Europe and the United Kingdom. This could be because of the fact that many longstanding and mature orthopedic and cardiac registries have recognized that PROMs can provide an additional layer of knowledge beyond what clinical data alone can provide,^12^ and reinforces the need to explore PROMs in breast, penile, and mesh registries in greater depth.

The present review revealed variation in PROMs used across registries, and within registry types. This lack of standardization, making comparison between different registries difficult. Although we recognize that it is important for PROMs to be validated, we did not explore the validity of the PROMs chosen by the registries, because we placed emphasis on the way the PROMs were used specifically in relation to the way they were analyzed, rather than focusing on the PROMs chosen. Most registries favored generic PROMs over disease-specific PROMs, for example, the EQ-5D and VAS, were the most frequently used by both orthopedic and cardiac registries. Generic PROMs have a greater applicability for use among many populations and are generally used to compare populations, and interventions at aggregate level.^97^ Disease-specific PROMs are considered to have greater responsiveness to clinical changes in patient symptoms with more precise estimates; however, they are suited to making comparisons within a population, but not for comparisons across populations.^98,99^ The strength of generic PROMs to allow comparison across different disease states may account for the widespread use of generic tools that was observed. It has been recommended that both generic and condition-specific PROMs be used concurrently to counteract the limitation of using one over the other.^97^ Nevertheless, for the purpose of comparing performance between groups or time points, for risk factor prediction, or for time-to-revision analyses, disease-specific PROMs would likely be most appropriate.

The most common study design utilized was comparison of groups (devices or procedures). This finding is interesting as the limitations of comparative studies for clinical registries, such as the lack of ability to adjust for unmeasured confounders and selective reporting, are well known.^100,101^ Observational registry studies cannot match the degree of comparability that a RCT can achieve unless specific analytical strategies have been employed: direct comparison, parallelization, subgroup analysis, matched pairs, outcome adjustment, or propensity scoring.^100^ The choice by registries to use comparative analysis for PROM data despite the statistical challenges may be offset by the large sample sizes that registries can obtain. Moreover, using registry data for comparison of outcomes may also be a way of studying important clinical questions, using real-life evidence, without having to undertake a prospective RCT which can be costly and only capture a highly selective, subset of patients (ie, those who consent, which is often only a fraction of patients, especially in surgery).^102-104^

Studies comparing time points, and studies for survival analysis were also identified in the literature, however, of particular importance were the predictive studies that we identified in keeping with the objective of the ABDR to identify poorer breast device performance.^22,71,72,75,88^

We observed a lack of papers utilizing PROMs as part of a predictive or risk factor model, through regression analysis. Specific to high-risk medical device, risk prediction analysis is necessary to establish if poor PROM outcomes are associated with implant failure and/or revision surgery and the degree to which we can be certain that such conclusions are accurate. In a study, Sharma et al highlighted that risk prediction models in front-line clinical practice remain underutilized.^105^ Although Sharma's paper considered front-line clinical settings and not registry settings, based on our findings, the same may be said for high-risk medical device registries. Just over half of these predictive papers analyzed PROMs as an outcome only and sought to understand what clinical factors affect PROM scores, and/or used regression to understand how early PROMs were associated with longer term PROMs.^21,33,40,48,51,61,83,90^

In 2 studies that treated PROMs both as a factor in regression analysis and as an outcome,^28,86^ both identified that patients with lower PROM scores were more likely to experience poorer outcomes. Ideally, when PROMs are analyzed in regression, the identification of problematic symptoms can alert surgeons and prompt further discussion and management. The predictive studies described a correlation between PROM scores and clinical outcomes following implant surgery.^22,71,72,75,88^ Although these studies sought to utilize the potential of PROMs in high-risk surgical registries, they have limitations. For example, the models used did not generally account for site or surgeon factors, and there was no evidence of sensitivity analysis for missing data, there was no consistent approach to optimal follow-up time points, and not all studies clearly reported their RRs. This can also create bias in the analysis, either under-estimating or over-estimating the effect size depending on the nature of the relationship between variables.^100,101^ Overall, all 5 predictive studies identified an association between postsurgical PROMs and outcomes to varying degrees. This is important as it shows that PROMs were good predictors of postsurgical outcomes or revision surgery, an important implication for surgeons looking to identify at-risk patients.

Our review revealed that PROMs were predominantly studied over short-term time points. The 5 papers that considered PROMs as a risk factor in relation to a clinical outcome looked at short-term outcomes, apart from Rothwell et al^75^ and Hosman et al,^72^ who considered time points within 26 months from time of device insertion. This may be because of insufficient PROM RRs and/or limited resources to collect PROMs for long-term follow-up. The temporality observed in the majority of papers suggest that from an orthopedic and cardiac context, the probability of an adverse event is most likely to occur in this short-term timeframe, and an early “poor” PROM may be a good indicator of complications and/or revision. It may also be feasible to infer that the further away PROM data are collected from the intervention, the greater potential there is for external confounders to impact on these, making longer term prediction problematic, as it may be difficult to assign causality because of other life events happening to patients. It is worth noting that the type of PROM and relevant time points would vary depending on which medical conditions the registry serves. Some high-risk devices, such as breast implants or pelvic mesh, have been recognized to have complications that may occur many years after insertion, such as breast-implant-associated anaplastic large cell lymphoma or chronic pain resulting from pelvic mesh breakdown.^6,106,107^ Thus, further research needs to be undertaken into the feasibility and sensitivity of long-term device monitoring with PROMs in these circumstances.

Variation in RRs was a finding that is worthy of a brief discussion given their relevance to the minimum RRs required by registries. Almost half of the publications provided limited information regarding RRs, where RRs were either not presented in the literature or not clear or readily available requiring calculation. This is consistent with papers by Wang et al^108^ and Ruseckaite et al^109^ who investigated PROM RRs in registry-based studies. They found a wide variation in RRs and how they were reported, if reported at all.^108,109^ Interestingly, few papers discussed the treatment of missing data or multiple imputations to account for the uncertainty of low RRs or nonresponse which may lead to biased inferences.^108^ As registries include a preselection of patients, equally important is the need for registries to disclose selection, inclusion, and capture rates, attrition, temporality of PROMs, patient denominator, and site type, that is, public vs private, all of which can skew the observed results. The impact of this is that the risks and benefits of surgery cannot be conveyed to surgeons and patients when nonresponse bias may be present.^110^ We recommend this be an important consideration for high-risk medical device registries when implementing PROM programs and in the reporting of PROM data.

We recognize that certain elements, such as the effect of opt-in vs opt-out, national vs regional PROM collection, and what determines PROM RRs may be of interest, however, were beyond the scope of this manuscript. We can report that no relationship between low capture rate and high PROM return vs a high capture and low RR was observed.

PROM collection has changed over the years, and electronic collection of PROMs has made an impact on the PROM landscape; interestingly, no marked difference was observed in RRs in PROMs collected prior to 2012. We were unable to locate information regarding clinically relevant starting points for PROM RRs; however, Wang et al^108^ suggest that many clinical registries aim for RRs of at least 80%; however, this was not achieved by the majority of studies in our review (Figure 6). Contrary to Wang et al,^108^ Johnson and Wislar^110^ mention an RR of 60% to be the threshold of acceptability as a measure of survey quality, though they acknowledging that this figure has not been scientifically proven (Figure 6). We recommended a framework for PROMs when reporting completeness, to underscore the validity of results in the absence of RRs above 80%. The inconsistent reporting of RRs in our study made it difficult to identify a trend and establish if an association between PROM time points and RRs exists. To influence the clinical decision-making for high-risk medical device applications, given the potential implications for device regulation and clinical decision-making, a high RR threshold is needed. Alternatively, if results are reported with a high degree of significance at a lower RR or if participant numbers are very high, it is not clear whether the correlation estimates can be considered precise enough for clinical decision-making. We recommend sensitivity analysis or multiple imputations be carried out to allow researchers and surgeons to estimate the impact of bias on the reported results. The ABDR is an example of a large data set with approximately 65,992 patients involved in the PROM program. The registry observed a decrease in RRs from 49% to 79% in 2018 to 33% to 47% in 2021.^111^ As a result, the registry is re-evaluating its PROM program and accounting for missing data at the analysis stage through methods described earlier in this paper.

An implication of this study is that the ABDR will use the knowledge to inform regression models and improve transparency when undertaking PROM analysis and reporting, providing surgeons with complete information for clinical decision-making.

To our knowledge, this was the first scoping review to consider PROMs in high-risk medical device registries. The purpose of this paper was to collect more information about high-risk medical device registries that do collect PROMs; therefore, articles were not collected on high-risk device registries that do not collect PROMs, and it may be possible that many high-risk device registries do not capture PROMs. We were unable to determine the proportion of high-risk device registries that collect PROMs based on the data that were collected.

## CONCLUSIONS

This review synthesized the literature on how PROMs have been used among high-risk medical device registries. We found evidence supporting the use of PROMs in high-risk medical device registries. The purpose that PROMs serve in high-risk medical device registries range from being used to compare outcomes between groups and time points, to being used as predictive tools by predicting adverse events and aiding clinical decision-making for surgeons. It is recommended for transparency that, all registry-based PROM studies report their RRs and review their missing data for evidence of bias. We identified only a few studies in high-risk medical device registries where PROM data were used for predictive purposes, and although these are promising, methodological considerations need to be addressed. Finally, most of the studies in medical device registries using PROMs conducted so far have analyzed short-to-medium term outcomes, which are beneficial to identifying early postoperative problems. This does not address the issue of whether PROMs can feasibly and appropriately be used to support monitoring of longer term complications arising from high-risk implantable devices such as breast implants or pelvic mesh. The ABDR will use the findings from this study to guide future statistical analysis and reporting of PROMs.

## Data Availability

The data that support the findings of this study are available from the corresponding author upon reasonable request.
